# Insights into the pharmacodynamics and pharmacokinetics of meldonium after exposure to acute high altitude

**DOI:** 10.3389/fphar.2023.1119046

**Published:** 2023-02-22

**Authors:** Fengying Liu, Xin Sui, Qian Wang, Jinglai Li, Weijie Yang, Yi Yang, Zhenyu Xiao, Yangyang Sun, Xiaoxuan Guo, Xinyi Yang, Jun Yang, Yongan Wang, Yuan Luo

**Affiliations:** ^1^ State Key Laboratory of Toxicology and Medical Countermeasures, Beijing Institute of Pharmacology and Toxicology, Beijing, China; ^2^ Guollence Pharmaceutical Technology Co., Ltd., Beijing, China; ^3^ School of Pharmaceutical Science, Liaoning University, Shenyang, China

**Keywords:** meldonium, high altitude, acute injury, pharmacokinetics, pharmacodynamics

## Abstract

**Objective:** Meldonium, a well-known cardioprotective drug, has been reported to be protective against pulmonary injury at high altitudes; however, the pharmacodynamics of meldonium in other vital organs under acute high-altitude injury are less investigated and the related pharmacokinetics have not been fully elucidated.

**Methods and Results:** The present study examined the basic pharmacodynamics and pharmacokinetics (PK) in rat exposure to acute high-altitude hypoxia after intragastrical and intravenous pre-administration of meldonium. The results indicate that meldonium can improve acute hypoxia-induced pathological damage in brain and lung tissues, and restore blood biochemistry and routine blood index of heart, liver and kidney tissues under a simulated acute high-altitude environment. Furthermore, compared to the normoxia group, rats exposed to simulated high-altitude hypoxia and premedicated with intragastrical meldonium showed linear kinetics in the dose range of 25–100 mg/kg, with a significantly increase in the area under curve (AUC) and reduced clearance rate. No significant differences in these meldonium of PK parameters were observed with intravenous administration. Additionally, meldonium was involved in the regulation of succinic acid and 3-hydroxypropionic acid.

**Conclusion:** These results will contribute to our understanding of the preclinical PK properties of meldonium and its acute high-altitude protective effects.

## Introduction

Over 140 million people worldwide travel to high-altitude regions for tourism, military, sport, or work, and acute hypoxia-induced injury has become a health hazard for lowland people who move rapidly to high altitudes (Basnyat & Murdoch, 2003). Sufficient evidence has shown that acute hypoxia induced by multiple environmental factors (temperature and humidity, oxygen concentration <18%) and pathological changes (impaired respiratory exchange) can lead to tissue and organ damage and even death (Schoene, 2008; Luks et al., 2017). Furthermore, the influence of high-altitude hypoxia on the respiratory system, cardiovascular system, and nervous system further lead to related sickness including high-altitude headache, acute mountain sickness (AMS), central sleep apnea syndrome, high-altitude cerebral edema (HACE), and high-altitude pulmonary edema (HAPE) (Baumgartner, 1999; Sanchez del Rio & Moskowitz, 1999; Woods & Alcock, 2021). Therefore, further investigation into the prevention and treatment of high-altitude-related illnesses requires urgent attention.

Blood rheology, blood biochemistry, and organ functions changes caused by acute hypoxia at high altitude may alter the pharmacokinetic of certain drugs. For example, changes in the number and composition of intestinal microorganisms in an alpine environment may lead to slower metabolic activity of nifedipine *in vivo*, thereby improving the bioavailability and therapeutic efficacy of nifedipine (Zhang et al., 2018). Acetaminophen, an antipyretic and analgesic drug, increased the elimination of time and area under the curve (AUC) and significantly reduced clearance rate (CL/F) in rats after exposure to a high-altitude environment (Zhu et al., 2021). Likewise, phenytoin plasma concentration and cerebrospinal fluid concentration were significantly increased under acute hypoxia (Zhang et al., 2020). At present, more attention focuses on the treatment of high-altitude illnesses and related mechanistic studies while there are few reports on the drug pharmacokinetics under acute hypoxia conditions.

Meldonium, as an energy regulator, blocks myocardial hypoxia by competitively inhibiting carnitine-dependent fatty acid oxidation and promoting anaerobic oxidation of the glycolytic pathway (Dambrova et al., 2016). In addition, it improves the utilization of acetyl-CoA by various mitochondrial metabolic pathways by inhibiting carnitine acetyltransferase, preventing a decrease of ATP and ADP concentrations, thereby exerting an energy optimization (Dambrova et al., 2002). Our previous studies have reported the protective effect of meldonium in high-altitude lung injury and relevant mechanisms (Wang et al., 2022). However, the basic efficacy and pharmacokinetics of prophylactic administration of meldonium for acute high-altitude injury has yet to be evaluated. We thus established a simulated acute high-altitude rat injury model to evaluate basic pharmacodynamic and pharmacokinetic indicators and to provide a reference for preventive medication and clinical studies of high-altitude illness.

## Materials and methods

### Chemicals, reagents and instruments

Meldonium (purity > 99.9%) was purchased from Yuanye Pharmaceutical Co. (Shanghai, China). L-carnitine (purity > 99.9%) was purchased from the National Institute for the Control of Pharmaceutical and Biological Products (Beijing, China). A stable isotope labeled internal standard compound, acetonitrile and methanol, were obtained from Merck Chemical Co., (Darmstadt, Germany). Formic acid and ammonium acetate were purchased from Thermo Scientific (Waltham, United States). All the other chemicals were of analytical grade or better.

The Liquid chromatography-tandem mass spectrometry (LC-MS/MS) system consisted of a liquid chromatograph (Waters Technologies, Massachusetts, United States) and coupled with an AB SCIEX 6500 triple quadrupole mass spectrometer (Sciex Corp., Framingham, United States). A FLYDWC50-1A animal simulated hypobaric hypoxia chamber was purchased from Guizhou Fenglei Oxygen Chamber Co., Ltd. (Anshun, China). An FLPI-2 laser doppler blood flow monitor (Moor Instruments, Axminster, UK), a high-content imaging analysis microscope (Molecular Devices, California, United States), an ABL90 Flex blood gas analyzer (Radiometer Medical, Copenhagen, Denmark), and an IX51 light microscope (Olympus, Japan) were also applied in this study.

### Animals

Specific pathogen-free (SPF) Sprague–Dawley (SD) rats weighing 200 ± 20 g were purchased from SIBEIFU Biotechnology Co., Ltd. (Beijing, China). SD rats were housed with an environmental temperature of 22°C ± 2°C and 40%–70% relative humidity. All the experimental animals were acclimated under the above conditions for 3 days and fasted overnight before the experiments. Operations for animal experiments were carried out the Guide for the Care and Use of Laboratory Animals (NBCDSER-IACUC-2018-096, Institutional Animal Care and Use Committee of Laboratory Animal Center, Beijing, China).

### Acute hypoxia injury model

To evaluate the effect of pre-administrated with meldonium under acute high-altitude hypoxia, SD rats (6 in each group) were administered by gavage and randomly divided into normoxia group, hypoxia group (model group), hypoxia groups with differential dose meldonium (25, 50, 100, 200, and 400 mg/kg), and hypoxia with acetazolamide group (positive control). In addition, the pharmacokinetics of meldonium (25, 50, 100 mg/kg) was investigated under acute high-altitude hypoxia in SD rats administered by gavage and/or intravenously. Normoxia group rats given saline lived at an average altitude of 43.5 m, and the acute hypoxia groups were placed in a simulated hypobaric hypoxia chamber with an altitude of 7,000 m for 24 h after 3 days of continuous administration with meldonium.

### Blood gas measurements

Rats were anesthetized upon exit from the hypobaric hypoxia chamber. Blood samples were drawn from the abdominal aorta into polypropylene syringes containing heparin and immediately transferred to a biochip to keep blood gases near physiological values. Acid-base balance, metabolites, and electrolytes changes, including blood bicarbonate (pHCO_3_^−^), pH, partial pressure of carbon dioxide (pCO_2_), partial pressure of oxygen (pO_2_), glucose (Glu), lactic acid (Lac), sodium (Na^+^), potassium (K^+^), and chloride (Cl^−^) were assessed using a blood gas analyzer (ABL90, Radiometer, Copenhagen, Denmark).

### Laboratory parameters analysis

The screening included an evaluation of vital functions (systolic and diastolic blood pressure, and mean arterial pressure), biochemical blood analysis, and routine blood examination, which were used for the initial assessment of hypoxia injury and efficacy of meldonium.

Initial assessment of liver injury included measuring levels of serum alanine and aspartate aminotransferases (ALT and AST). Serum creatinine acid (CREA) and blood urea nitrogen (BUN) were used as indicators for kidney damage. Creatine kinase (CK) and serum lactate dehydrogenase (LDH) were used as biochemical markers of cardiac injury. Complement C3 and C4, involved in the immune response, were also detected. In addition, red blood cells (RBC), white blood cells (WBC), hemoglobin, and lymphocytes (LYMPH) were evaluated.

### Histopathological evaluations(H&E)

Twenty-four hours post simulated high-altitude hypoxia exposure, rat brain tissue was perfused with 0.9% normal saline and 4% paraformaldehyde solution. The brain tissue was removed, fixed, dehydrated by graded ethanol, embedded in paraffin, and cut into 5 μm thick sections. Sliced sections were stained with hematoxylin and eosin (H&E) and examined using an optical microscope (XDS-113, Olympus Corporation, Japan).

### Doppler monitoring of blood flow

Relative cortical cerebral blood flow was measured with a laser Doppler probe secured to the dura matter. Rats were euthanized, and the skull was exposed by cutting the skin of the head and washed with 150 µL saline. Blood flow in cerebral vessels was captured continuously by low-frequency pulsed ultrasound using laser doppler flowmeters (Moor, England). Results were recorded and analyzed by Moor FLPI-2 Review V50 software.

### LC-MS/MS conditions and pharmacokinetics

High-performance liquid chromatography (HPLC) and mass spectrometer analyses of meldonium were performed on a Thermo Hypercarb C18 column (3 mm × 100 mm, 5 μm, Thermo Fisher Scientific, Germany). Autosampler temperature was maintained at 15°C and the injection volume was 5 μL. The flow rate was set at 500 μL min^-1^. The mobile phase consisted of methanol and 5 mM ammonium acetate buffer solution containing 0.1% formic acid. The mass spectrometer was operated in the positive ESI mode with a drying gas temperature of 350°C, nebulizer pressure of 40 psi, capillary voltage of 4000 V, and multiple reaction monitoring (MRM) for meldonium with scan time100 m/s (m/z, 147.1 → 58.2).

To explore the pharmacokinetics of meldonium under acute high-altitude hypoxia, 0.2 mL of venous blood was collected in an anticoagulation tube (EDTA-K2) before (time 0) and at 0.08, 0.25, 0.5, 1, 2, 4, 8, 12, and 24 h post meldonium administration, and then centrifuged at 2,500 *g* for 15 min at 4°C. The blood collection time for *i.v.,* administration was increased by 2 min.

Rat liver homogenate, as the incubation system, was taken and added 100 μL of meldonium working solution concentrations (400 μg/mL, 200 μg/mL, 100 μg/mL, 40 μg/mL, 20 μg/mL, 10 μg/mL, and 4 μg/mL) to vortex and mix, and placed in a 37°C water bath for incubation. The samples supernatant was taken at 0 min, 15 min, 30 min, 1 h, 2 h and 4 h, respectively, for determination in LC-MS/MS.

### Statistical analysis

Pharmacokinetic parameters of meldonium including peak time (T_max_) and peak concentration (C_max_), area under curve (AUC_all_) of plasma concentration-time, the apparent volume of distribution (V_z_/F), clearance rate (CL/F), and last mean residence time (MRT_last_) were calculated using Phoenix WinNonlin software 8.0 (Certara, Princeton, United States). Data are expressed as mean ± SEM. Statistical analyses were performed using GraphPad Prism 6 (GraphPad Software, La Jolla, US) and assessed by the one-way ANOVA followed by Tukey’s test. *p* < 0.05 were considered statistically significant.

## Results

### Behavioral investigation

**FIGURE 1:**
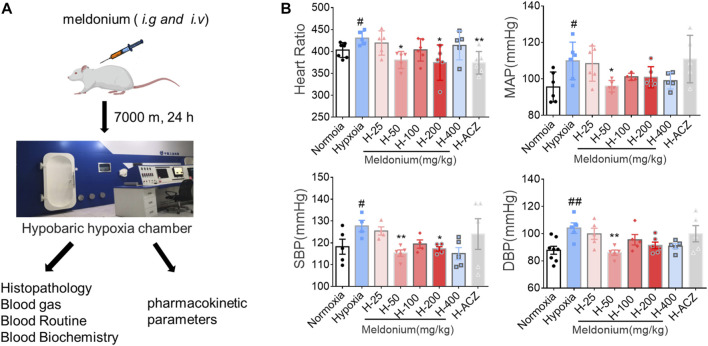
Simulation of acute high-altitude hypoxia injury rat model designed and evaluated *in vivo*. **(A)** Experimental design of simulated acute hypobaric hypoxia injury in Sprague Dawley rats. **(B)** Heart rate and blood pressure measurements (n = 6, mean ± SEM). ^#^ *p* < 0.05 and ^##^ *p* < 0.01 compared to the plain normoxia group; ^*^ *p* < 0.05 and ^**^ *p* < 0.01 compared to the high-altitude hypoxia with saline group. The data were analyzed using one-way ANOVA analysis of variance with Tukey’s post-tests. H-25, H-50, H-100, H-200, H-400, indicate different doses of meldonium under hypoxia, respectively, and H-ACZ indicates the administration of acetazolamide under hypoxia.

SD rats were exposed to simulated high-altitude environment for the hypoxia test, and the normoxia group was used as parallel control (Figure 1A). Within the first 15 ∼ 20 min of increasing altitude by 30 m/s, compared with the normoxia group, increased activity frequency, faster abdominal breathing, and head-up posture and neck extension were observed in the hypoxia group. With prolonged periods of hypoxia, activity frequency of rats gradually decreased. When reaching the specified altitude of 7,000 m, rat huddled together, and trembling continued until the end of hypoxia treatment. This hypoxia model can induce abnormal activities or affect rat behavior.

By comparison, we found that heart rate tended to increase under acute hypoxia compared to the normoxia group, while heart rate in the meldonium (50 mg/kg and 200 mg/kg) groups were significantly lower after prophylactic administration (*p* < 0.05). We then compared changes in systolic blood pressure (SBP), diastolic blood pressure (DBP), and mean arterial pressure (MAP) in normoxia and hypoxia group as well as after meldonium treatment and found a significant increase in SBP, DBP, and MAP in the hypoxia group (*p* < 0.05). Further, SBP, DBP, and MAP values in the meldonium pretreatment group by *i.g.,* administeration (50 mg/kg) significantly improved (*p* < 0.05) and were superior to the positive control drug acetazolamide (Figure 1B).

### Pathological, physiological and biochemical examinations

#### Hematoxylin-eosin staining

**FIGURE 2:**
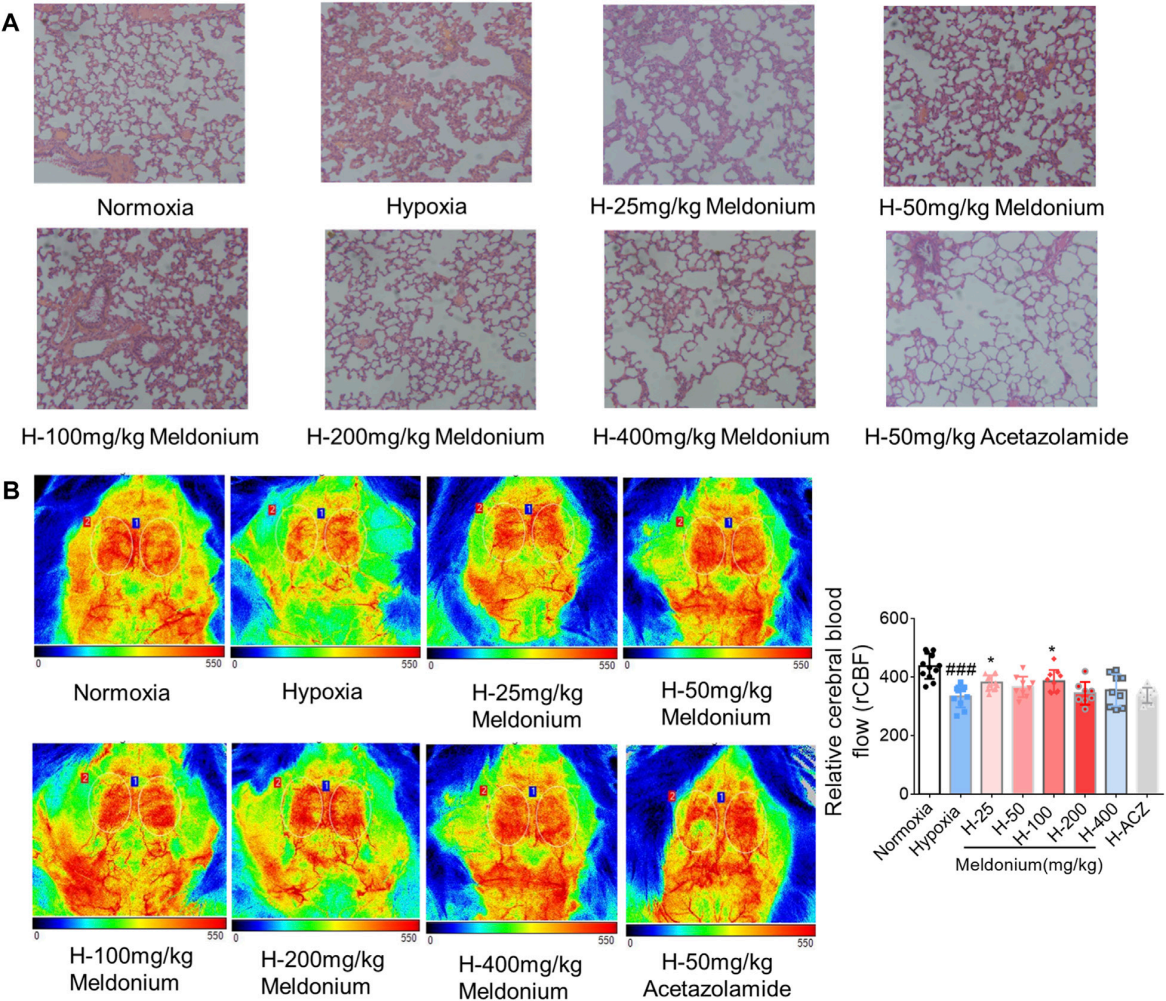
Representative images showing pathological effects in the absence and presence of meldonium on brain and lung tissue injury under acute high-altitude hypoxia. **(A)** Representative figures of lung tissue using HE staining (200×). **(B)** Quantitative and representative doppler blood flow map (n = 6, mean ± SEM). ^###^ *p* < 0.001 compared to the normoxia group; ^*^ *p* < 0.05 compared to the high-altitude hypoxia with saline group. The data were analyzed using one-way ANOVA analysis of variance with Tukey’s post-tests. H-25, H-50, H-100, H-200, H-400, indicate different doses of meldonium under hypoxia, respectively, and H-ACZ indicates the administration of acetazolamide under hypoxia.

High-altitude environments affect heart and lung function (Mallet et al., 2021). As shown in Figure 2A, compared with the normoxia control group, alveolar dilatation, alveolar interstitial fracture and thickening, and disorganized cell arrangement were observed in the acute hypoxia injury group. However, compared with the hypoxia group, alveolar dilatation, alveolar fracture and thickening, and cell arrangement were significantly reduced in the meldonium *i.g.,* administration (25 mg/kg, 50 mg/kg, and 100 mg/kg) groups; other meldonium *i.g.,* administration (200 mg/kg and 400 mg/kg) groups exhibited only improved alveolar interstitial thickening. Similarly, the positive control drug acetazolamide reduced the degree of alveolar interstitial thickening under hypoxia without significantly improving alveolar dilatation, alveolar interstitial disruption and cellular disorganization.

#### Cranial doppler flow analysis

Brain injury is another issue associated with high altitude (Ma et al., 2019). To examine the influence of acute hypoxia-induced cerebral ischemia injury, laser doppler blood flowmetry was used to determine regional cerebral blood flow in rats. As shown in Figure 2B, the hypoxia group rats exhibited a 23.6% decrease in rCBF, compared to the normoxia group (*p* < 0.01), while meldonium *i.g.,* administration groups (25 mg/kg and 100 mg/kg) significantly restored these brain blood changes and was superior to acetazolamide group.

#### Blood gas, electrolytes, and metabolites

**FIGURE 3:**
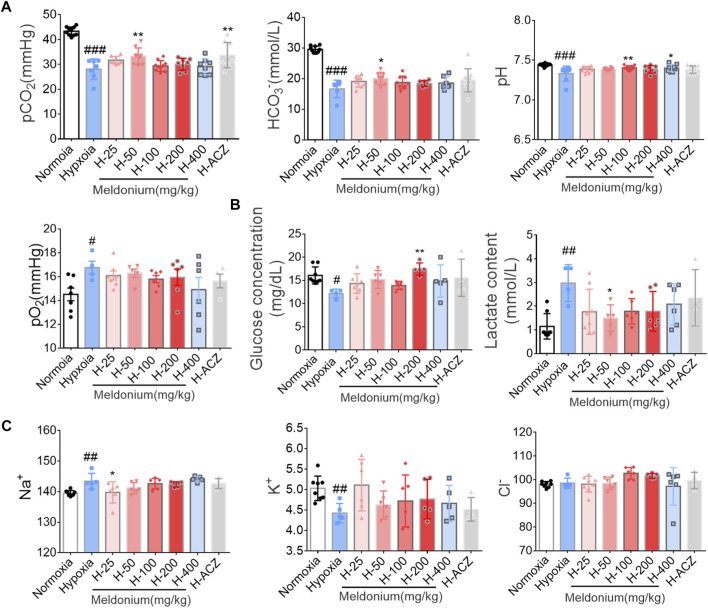
Effect of meldonium on arterial blood gas analysis under hypoxia injury. **(A)** Acid-base balance parameters; **(B)** Metabolites; **(C)** Electrolytes. Data are presented as the mean ± SEM (n = 6). ^#^ *p* < 0.05, ^##^ *p* < 0.01 and ^###^ *p* < 0.001 compared to the normoxia group; ^*^ *p* < 0.05 and ^**^ *p* < 0.01 compared to the high-altitude hypoxia with saline group. The data were analyzed using one-way ANOVA analysis of variance with Tukey’s post-tests. H-25, H-50, H-100, H-200, H-400, indicate different doses of meldonium under hypoxia, respectively, and H-ACZ indicates the administration of acetazolamide under hypoxia.

After exiting from the hypobaric hypoxia chamber and descending to the plain, blood samples were collected immediately from the abdominal aorta to examine the changes in blood gas, electrolytes, and metabolic values. Blood pH, pCO_2,_ and HCO_3_^−^ in the hypoxia group was significantly decreased compared with the normoxia group after acute hypoxia (Figure 3A). Pretreatment with meldonium (50 mg/kg) significantly upregulated the above indicators. However, pO_2_ levels were elevated in the hypoxia group compared to the normoxia group, and there was no significant difference in the pre-administered meldonium groups. During acute hypoxia, the body is involved in a defensive glycolytic process in which glucose is broken down to lactate. As shown in Figure 3B, lactate content was significantly elevated in the hypoxia group, while pre-administered meldonium group (50 mg/kg) significantly inhibited excessive lactate production. In addition, sodium levels were significantly higher, potassium levels were significantly lower, and chloride levels were not significantly different after acute hypoxia. 25 mg/kg dose of meldonium *i.g.,* administration group significantly reduced sodium levels (Figure 3C).

#### Routine blood examination and biochemical analysis

**FIGURE 4:**
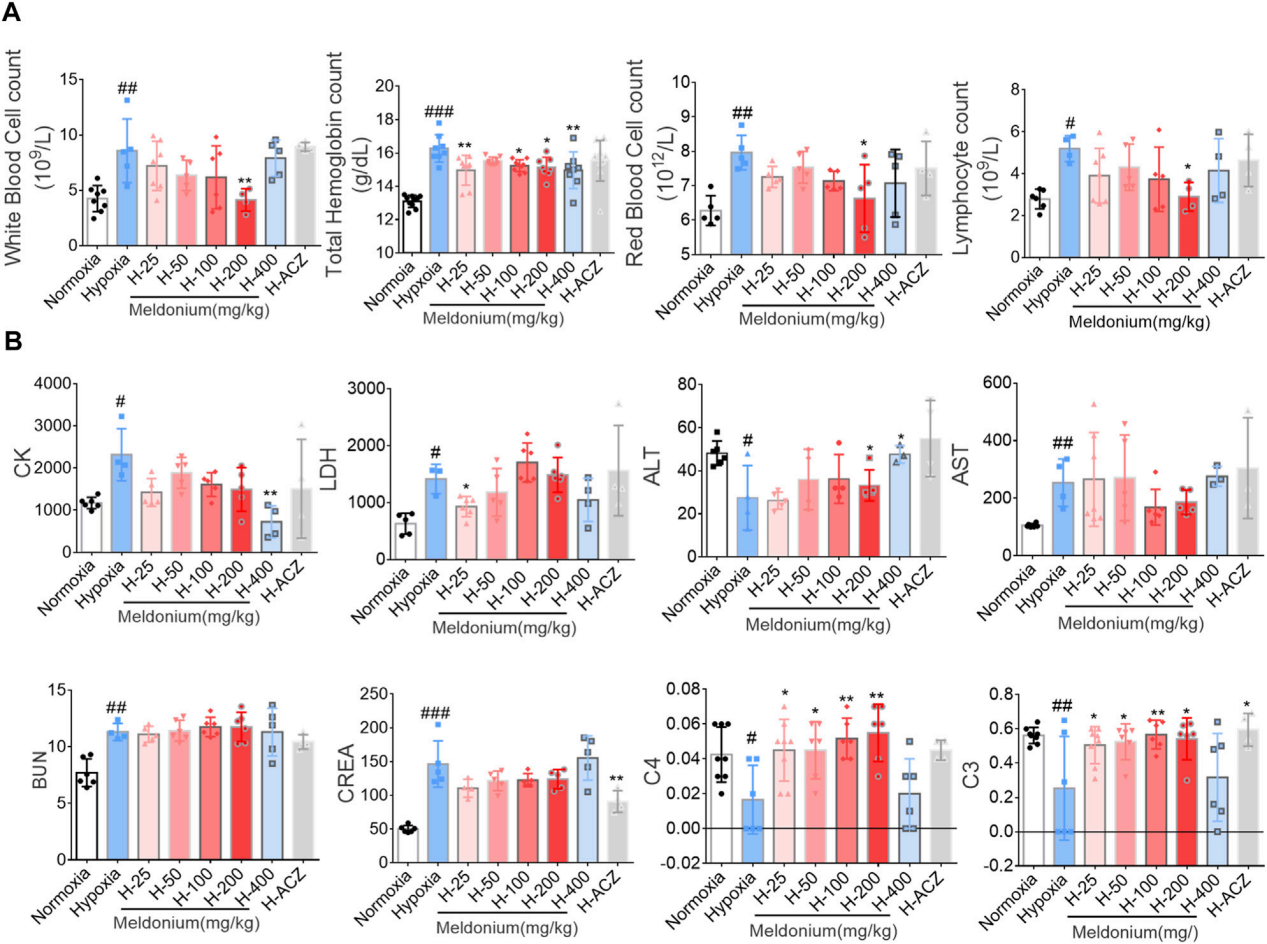
Effect of meldonium on pharmacodynamic blood tests under acute hypoxia injury. **(A)** Routine blood examination; **(B)** Biochemical analyses. Data are presented as the mean ± SEM (n = ). ^#^ *p* < 0.05, ^##^ *p* < 0.01 and ^###^ *p* < 0.001 compared to the normoxia group; ^*^ *p* < 0.05 and ^**^ *p* < 0.01 compared to the high-altitude hypoxia with saline group. The data were analyzed using one-way ANOVA analysis of variance with Tukey’s post-tests. H-25, H-50, H-100, H-200, H-400, indicate different doses of meldonium under hypoxia, respectively, and H-ACZ indicates the administration of acetazolamide under hypoxia.

To determine the effects of meldonium on cardiac, hepatic and renal injury under acute high-altitude conditions, Routine Blood Examination and Biochemical Analysis were assessed. Visible hemolysis arterial blood samples were excluded. At the cellular-level, compared to the normoxia group, acute hypoxia increased levels of WBC, RBC, LYMPH, and hemoglobin (*p* < 0.05), whereas pretreatment with meldonium group (200 mg/kg) restored the changes in WBC, RBC, and LYMPH numbers. 25–400 mg/kg dose group of meldonium showed significantly higher hemoglobin levels under acute hypoxia (Figure 4A). In addition, acute hypoxia injury elevated levels of biochemical parameters (CK, CREA, BUN, AST, and LDH) and decreased levels of ALT, C3 and C4. The hypoxia pretreatment group (400 mg/kg) reversed these alters in CK and ALT (*p* < 0.05), and in 25–200 mg/kg meldonium dose group showed significantly increased levels of complement C3 and C4, but meldonium did not significantly alter BUN and CREA (Figure 4B).

### Method validation

The methodological validation of meldonium in rat plasma is shown in Table 1. High levels of meldonium linearity were achieved within a range of 0.5–50 μg/mL (*r* ^2^ = 0.9960) with the lower limit of quantification at 0.5 μg/mL. Inter-batch precision and accuracy were determined by measuring six replicates of quality control (QC) samples at three concentration levels in rat plasma. Results show that the intra-batch precision of meldonium was less than 5.57%, inter-batch precision was less than 8.06%, and accuracy was less than 15%. The recoveries of internal standard calibration in rat plasma at three concentration levels of meldonium ranged from 99.07% to 105.28%. For ionization, the total relative standard deviations of the internal standard normalized matrix effect factors corrected for internal standard at low and high concentrations of meldonium were 4.84% and 4.64%, respectively, suggesting that there was no measurable matrix effect that interfered with meldonium determination in rat plasma. The meldonium solution was stable at room temperature.

**TABLE 1:** Validation parameters of meldonium’s biological method.

Parameter	Value
Correlation coefficient of calibration curve	*r* ^*2*^ = 0.9960
Range of calibration curve	500–50,000 ng/mL
Accuracy deviation	−15.27%–1.18%
Intraseries precision	1.03%–5.57%
Interseries precision	4.63%–8.06%
Normalized matrix effect factor	Coefficient of variation of matrix factor: 4.84% and 4.64%
Matrix selectivity (mean interference rate)	1.71%
Stability test at 4°C (relative deviation)	7 days: 1.19% ∼ 3.45%; 21 days: 2.25% ∼ 2.27%
Stability test with freeze–thaw at −20°C	7 days: 91.6%–95.19%; 21 days: 81.8%–89.01%
Stability test at room temperature	4 h: 94.32%–96.9%
Carryover effect	≤20% of LLOQ value
Rate of recovery	99.07% ∼ 105.28%

### Pharmacokinetics

**FIGURE 5:**
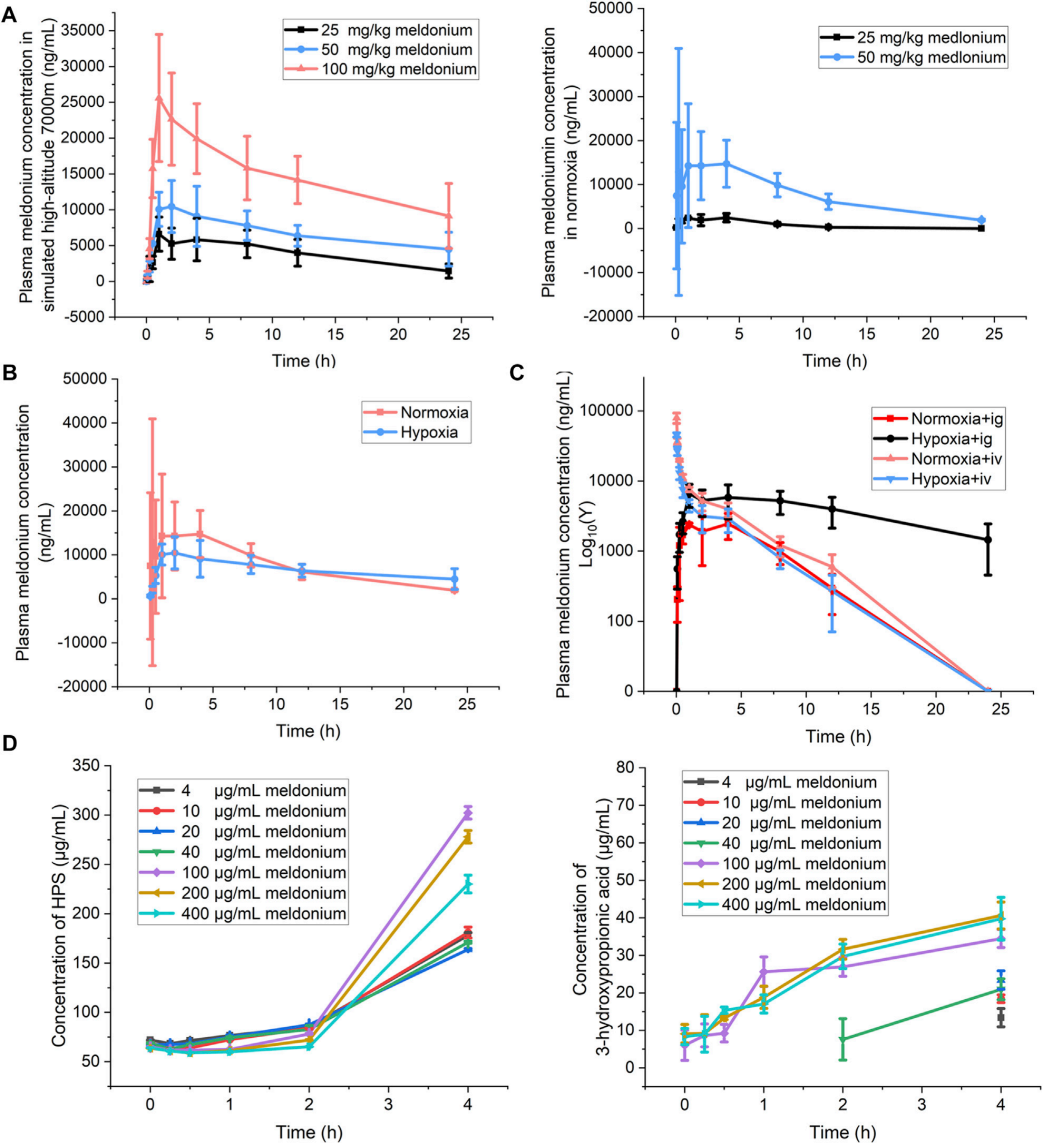
Detection of parameters related to meldonium pharmacokinetics. **(A)** Drug concentration-time curves of meldonium content in serum after *i.,g.* administration between normoxia groups (25 and 50 mg/kg) and high-altitude groups (25, 50, and 100 mg/kg, n = 6); **(B)** Comparison of drug concentration time profiles of meldonium (50 mg/kg) after *i.g.,* administration in high-altitude and normoxia groups (n = 3); **(C)** Drug concentration-time curves of meldonium (25 mg/kg) after *i.v.,* and *i.g.,* administration between high-altitude and normoxia groups (n = 3); **(D)** The 3-hydroxypropionic acid and succinic acid content of meldonium after incubation in liver homogenates (n = 3). The data were analyzed using one-way ANOVA analysis of variance with Tukey’s post-tests.

Based on a preliminary pharmacodynamic examination, three dose groups of meldonium (low dose, 25 mg/10 mL/kg; medium dose, 50 mg/10 mL/kg; high dose, 100 mg/10 mL/kg) were selected for pharmacokinetic studies. Figure 5A show mean plasma concentration time profiles for meldonium. The normoxia dose groups showed that the AUC of meldonium administered by gavage exhibited non-linear clearance in the range of 25–50 mg/kg. However, concentration-time curves after exposure at 7,000 m high-altitude for 24 h showed an AUC of meldonium with a dose-dependent increase within the range of 25–100 mg/kg by intragastrical administration. Plasma C_max_, AUC_all_, and MRT_last_ in meldonium low dose group (25 mg/kg) by intragastric administration under hypoxia showed significantly higher than those of the same dose normoxia group (Table 2).

**TABLE 2:** Pharmacokinetic parameters of meldonium in rat blood plasma between normoxia and hypoxia.

PK parameters	Normoxia	Normoxia	Hypoxia	Hypoxia	Hypoxia
	25 mg/kg meldonium	50 mg/kg meldonium	25 mg/kg meldonium	50 mg/kg meldonium	100 mg/kg meldonium
T_max_ (h)	3.00 ± 1.73	3.04 ± 1.58	4.33 ± 3.14	1.83 ± 1.17	1.33 ± 0.52
C_max_ (ng/mL)	2,709.92 ± 649.7	24,904.97 ± 22,618.59	7,321.69 ± 2060.91^a^	11,118.71 ± 3,515.13^a^	25,761.42 ± 8,678.83
AUC_all_ (h*ng/mL)	17,415.21 ± 3,925.96	171,805.85 ± 36,723.18	86,443.85 ± 39,554.66^a^	162,116.00 ± 39,767.98	329,712.29 ± 115,319.66
Vz/F (mL/kg)	5,335.80 ± 1,286.73	2,773.03 ± 756.42	3,302.48 ± 1,139.24	5,032.12 ± 1,679.62^a^	4,773.61 ± 1,194.83
CL/F (mL/h/kg)	1,400.32 ± 362.55	251.50 ± 56.98	230.76 ± 149.96^a^	182.54 ± 76.49	170.40 ± 72.21
MRT_last_ (h)	4.34 ± 0.58	7.43 ± 1.25	8.40 ± 1.36^a^	10.20 ± 1.29	9.09 ± 1.79

^a^ Note: *p* < 0.05 compared to the normoxia group.

T_max_, time taken to attain maximum concentration.

C_max_, Maximum concentration achieved in the blood.

AUC_all_, area under concentration-time curve for the period from 0 min to infinity time.

Vz/F, the apparent volume of distribution.

CL/F, clearance rate.

MRT_last_, last mean residence time.

In order to more visually examine the pharmacokinetic changes of meldonium under normoxia and acute plateau hypoxia, we selected a medium dose of meldonium by intragastric administration for further investigation. As shown in Figure 5B and Table 2, compared to the normoxia group, reduced C_max_ (24,904.97 ± 22,618.59 Vs. 11,118.71 ± 3,515.13 mL/h/kg, *p* < 0.05) and increased Vz/F (2,773.03 ± 756.42 Vs. 5,032.12 ± 1,679.6 mL/kg, *p* < 0.05) of meldonium in plasma under acute high-altitude conditions were observed. In addition, the pharmacokinetics of low-dose intravenous and *i.g.,* administration under normoxia and acute high-altitude hypoxic conditions were evaluated (Figure 5C; Table 3). Similar to clearance of the medium dose of meldonium, plasma clearance for low-dose meldonium (25 mg/kg) given by *i.g.,* administration was significantly lower (1,400 ± 362 Vs. 230 ± 150 mL/h/kg, *p* < 0.05) and MRT_last_ was prolonged (4.34 ± 0.58 Vs. 8.40 ± 1.36 h, *p* < 0.05) under acute high-altitude hypoxia, compared to the normoxia group. However, no significant changes in these PK parameters were observed after low-dose intravenous administration, with comparable MRT_last_ (3.14 ± 0.68 Vs. 3.43 ± 1.44 h) and clearance (487 ± 34 Vs. 743 ± 252 mL/h/kg, *p* < 0.05) for normoxia and to high-altitude groups. Therefore, the pharmacokinetics of the different meldonium administration methods differ significantly under acute high-altitude hypoxia.

**TABLE 3:** Pharmacokinetic parameters of meldonium (25 mg/kg) administered by i.v., and i.g., Under acute high-altitude hypoxia.

PK parameters	Normoxia *i.v*.	Normoxia *i.g.*	Hypoxia *i.v.*	Hypoxia *i.g*.
	Administration	Administration	Administration	Administration
T_max_ (h)	0.03 ± 0.00	3.00 ± 1.73	0.03 ± 0.00	4.33 ± 3.14
C_max_ (ng/mL)	79,664.17 ± 13,493.34	2,709.92 ± 649.7	45,577.47 ± 3,199.05	7,321.69 ± 2060.91^a^
AUC_all_ (h*ng/mL)	48,999.24 ± 3,131.37	17,415.21 ± 3,925.96	28,920.63 ± 4,222.44^a^	86,443.85 ± 39,554.66^a^
Vz/F (mL/kg)	2,613.66 ± 1,368.78	5,335.80 ± 1,286.73	4,702.79 ± 1814.47^a^	3,302.48 ± 1,139.24
CL/F (mL/h/kg)	487.47 ± 34.66	1,400.32 ± 362.55	743.44 ± 251.94	230.76 ± 149.96^a^
MRT_last_ (h)	3.14 ± 0.68	4.34 ± 0.58	3.43 ± 1.44	8.40 ± 1.36^a^

^a^ Note: *p* < 0.05 compared to the normoxia group.

T_max_, time taken to attain maximum concentration.

C_max_, Maximum concentration achieved in the blood.

AUC_all_, area under concentration-time curve for the period from 0 min to infinity time.

Vz/F, the apparent volume of distribution.

CL/F, clearance rate.

MRT_last_, last mean residence time.

To investigate the *in vitro* metabolite of meldonium, succinate and 3-hydroxypropionic acid, rat liver homogenates with serial concentrations of meldonium were incubated. The study results showed that the metabolite 3-hydroxypropionic acid showed an increasing trend with concentration and time in the high concentration group (100–400 μg/mL), while no increasing trend was found with increasing content in the low concentration group (4–40 μg/mL), but an increasing trend was found after 4 h of incubation. The metabolite succinic acid showed an insignificant trend with concentration, but showed an increasing trend with increasing incubation time (Figure 5D).

## Discussion

Although insights into high-altitude sickness have made progress, there remains a scarcity of drugs for clinical application. Furthermore, the effects of acute high-altitude hypoxia at the tissue and organismal levels need to further investigation. Previous research has explored the protective effect of meldonium in acute high-altitude lung injury; however, whether pretreatment with meldonium has a protective effect on other organs at high altitude remains elusive. In this study, a low-pressure and low-oxygen simulation chamber was used to establish an acute rat injury model, and acetazolamide was used as a positive control drug. The study showed that meldonium (50 mg/kg) could significantly reverse heart rate and blood pressure changes, increase cerebral blood flow, alleviate symptoms of damaged lung tissues and improve the abnormal blood biochemistry in cardiac, hepatic and renal tissues in rats. In addition, we showed that acute hypoxia-induced altered blood concentrations of meldonium, resulting in a significantly longer elimination time and decreased in clearance. Notedly, meldonium regulates succinate, an important metabolic intermediate product of the tricarboxylic acid cycle (TCA). These data provide guidance for the clinical use of meldonium in future.

High-altitude environments, accompanied by the typical characteristics of hypobaric hypoxia, strong ultraviolet radiation, low temperature and high humidity, can easily induce pathological damage to various tissues, and in severe cases, can develop into brain or pulmonary edema. Clinical symptoms include headache, light-headedness, insomnia, fatigue and other neurological symptoms, as well as cough, progressive dyspnea and other signs of lung injury (Archer et al., 2020; K; Wang et al., 2018; Wu et al., 2018; Zafren et al., 2017). Pathological results show that meldonium can significantly improve cerebral blood flow reduction, alveolar dilatation, alveolar interstitial rupture and thickening induced by acute hypoxia. Acute hypobaric hypoxia environments induce metabolic acidosis, in which symptoms manifest as a result of a decrease in the levels of bicarbonate and carbon dioxide in the blood, while causing excessive production of lactic acid, and thereby an acid-base imbalance in the body (Zouboules et al., 2018; Caldwell et al., 2021). The 25 mg/kg meldonium dose group improved these changes. However, there was a significant increase in pO_2_ compared to the normoxia group, which possibly due to excessive oxygen uptake and enhanced oxygen permeability when removed from the simulation of hypobaric hypoxia chamber. Electrolytes act as an important buffering system to maintain acid-base balance in the body; meldonium is not sensitive to electrolyte changes under acute hypoxia.

Routine blood examination and biochemical analysis are an important part of high-altitude sickness diagnosis and treatment. Red blood cells and hemoglobin, as key components of transport and oxygen-carrying capacity, increase significantly in response to acute hypoxia; however, overproduction increases cardiac load and triggers cerebro-pulmonary circulatory disorders. Meldonium can improve changes in this index. In addition, the inflammatory response of the body under acute hypoxia results in a much higher than normal number of leukocytes, which can be suppressed by meldonium. Due to changes in tissue morphology and infiltration of inflammatory cells, especially with the decrease in energy levels, the immunity of the body is lower under acute hypoxia exposure. From biochemical tests assessing the efficacy of meldonium in cardiac, hepatic, renal and immune functions, it appears that meldonium is more effective in improving cardiac, hepatic and immune functions.

Hypoxia environments affect the intestinal flora and tissue injury, which in turn affects drug absorption, distribution, metabolism and excretion (Li et al., 2015; Pan et al., 2022). Both *i.g.,* and *i.v.,* drug administration achieved high serum concentrations and were generally well tolerated. We selected appropriate doses and administration modes based on the results of a preliminary pharmacodynamic study to further investigate the changes in pharmacokinetic-related parameters of its exposure to high altitude. The results of blood concentration measurements showed that absorption of meldonium in low dose (25 mg/kg) by intragastric administration increased significantly and excretion rate slowed with exposure to acute hypoxia, resulting in significantly higher plasma drug concentrations *in vivo* than in the normoxia group. Interestingly, there may be dose saturation with medium dose 50 mg/kg using intragastric administration of meldonium, resulting in a significantly lower C_max_ under acute hypoxia than in the normoxia group. In addition, CL in low-dose meldonium (25 mg/kg) group using intragastric administration at normoxia group (1,400.32 ± 362.55 mL/h/kg) was much higher than medium-dose meldonium (251.50 ± 56.98 mL/h/kg), suggesting a non-linear clearance in the range of 25–50 mg/kg dose in rats at normoxia, which is different from other Chinese pharmacokinetic data in healthy subjects (plasma AUC: 46–60 μg/mL·h; CL: 9.3–10.8 L/h after intravenous administration of 500 mg meldonium) (Peng et al., 2010; Zhao et al., 2016). This suggests that there may be species-dependent differences in clearance rats between rats and humans and appropriate adjustments should be made in the future dose administration of meldonium for human use.

Changes in liver function can impact metabolism of drugs in the body. In acute hypoxia, the body is metabolically imbalanced and provides energy and counteracts hypoxia by increasing glycolysis or promoting tricarboxylic acid cycle. Yoshisue found that the possible metabolites of meldonium in plasma are succinic acid and 3-hydroxypropionic acid, which was consistent with present study results (Yoshisue et al., 2000). This further validates that meldonium exerts its protective effect through energy metabolic pathways.

Considerable progress has been made in research on the treatment and mechanisms of high-altitude sickness; however, researches on rational use and preventive efficacy at high altitudes are scarce. In this study, the pharmacodynamics and pharmacokinetics of meldonium pre-administered exposure to acute-hypoxia environments were explored and a novel drug prevention strategy and clinical research in acute high-altitude injury was provided.

## Data Availability

The original contributions presented in the study are included in the article/Supplementary Material, further inquiries can be directed to the corresponding authors.
